# Shape and Boundary Similarity Features for Accurate HCC Image Recognition

**DOI:** 10.1155/2017/3764576

**Published:** 2017-11-07

**Authors:** Xiaoyu Duan, Huiyan Jiang, Siqi Li

**Affiliations:** Software College, Northeastern University, Shenyang 110819, China

## Abstract

Nucleus morphology is of great importance in conventional cancer pathological diagnosis, which could provide information difference between normal and abnormal nuclei visually. Therefore, this paper proposes two novel kinds of features for normal and hepatocellular carcinoma (HCC) nucleus recognition, including shape and boundary similarity. First, each individual nucleus patch with the fixed size is obtained using center-proliferation segmentation (CPS) method. Then, nucleus shape library is constructed based on manual selection by pathologists, which is utilized to measure nucleus shape similarity via Dice, Jaccard, precision, and recall coefficients. Meanwhile, boundary similarity is evaluated through triangles composed of some boundary feature points for each nucleus. Finally, the conventional random forest (RF) is used to train and test the classification model for HCC nucleus recognition. Extensive cross-validation tests could facilitate the selection of the optimal feature set and the experiment comparison results demonstrate that our proposed morphological features are more beneficial for classification compared with other traditional characteristics.

## 1. Introduction

Cancer is a leading cause of death in the world. In particular, in less developed countries, liver cancer is the second most common cancer compared to other cancers, in which the majority of primary liver cancer arises from liver cells and is called hepatocellular carcinoma (HCC). Throughout the treatment of liver cancer, probability of success cure will be hugely increased in the early stages. However, symptoms of early liver cancer are not obvious for patients and doctors to discover. Thus, early detection and diagnosis are of great significance for decreasing the mortality of HCC effectively.

Generally, a common method to confirm the diagnosis of HCC is through needle biopsy, which extracts some cells or a small piece of tissue from the affected area of the liver for analysis under a microscope. However, this diagnosis process is subjective, laborious, and time-consuming for operators. As is well-known, diagnosis from pathology images remains the “gold standard” for most cancers [1]. Therefore, the computer-aided diagnosis (CAD) for pathology image analysis has become a research hotspot in which the recognition of nucleus is regarded as a prerequisite. The accurate classification results could provide objective quantitative evaluations and facilitate the final diagnosis.

With the development of machine learning, several CAD models have been developed for pathology image process, which mainly include three parts, nucleus segmentation, feature extraction, and cell classification. For nucleus segmentation, Jung et al. [2] addressed the overlapped nuclei with an unsupervised Bayesian classification scheme. Distance transform, topographic surface, and the expectation-maximization (EM) algorithm were employed and the regular shape of clumped nuclei was viewed as a priori knowledge. Vink et al. [3] proposed an efficient nucleus detector which was merged by a large feature set and modified AdaBoost using a globally optimal active contour algorithm. The method improved the computational efficiency and also refined the border of the detected nuclei. In feature extraction stage, Huang and Lai [4] used a dial morphological grayscale reconstruction to achieve the accuracy of nuclear shapes. Fourteen features were extracted and a SVM-based decision-graph classifier was proposed for HCC classification. Liu et al. [5] regarded moment, Daubechies wavelets, and Gabor wavelets as three features of vital importance for the classification of cells. As for cell classification, Lorenzo-Ginori et al. [6] proved that cell classification just in the characteristics of nucleus could come into effect as well. A combination between morphological characteristics and Haralick texture features was obtained from the nucleus' gray-level cooccurrence matrix. A new heuristic search algorithm, Maximum Minimum Backward Selection (MMBS) was proposed in [7]. The Weighted Discernibility of Feature Subsets (WDFS) evaluation criteria were defined as the evaluation strategy of MMBS to solve the unbalanced samples, which contributed to a better feature subset. The experiment results showed a good classification performance for liver pathological image.

Recently, Gautam and Bhadauria [8] used four features of white blood nuclei and then some values of each feature, which were maximum and minimum, extracted for every class of white blood class. If the value of features for particular nucleus lays between the maximum and the minimum value of features values stored for particular class, then the segmented nucleus belonged to that class. Qi et al. [9] extracted 128-dimensional SIFT features from thousands of large patches which were densely sampled in multiple scales and were called RootSIFT. PCA was applied to the RootSIFT and IFV encoding was applied to the PCA-after features with prelearned GMM parameters for a better classification result. Xia et al. [10] defined three atypia features and provided some shape features, fractal dimension features, several gray features, and Tamura features. By using a HCC image classification model based on random forests and combined with VRRF, the method showed a good performance. Gallegos et al. [11] proposed an alternative method called feature subset selection (FSS). Feature subset selection (FSS) helped to decrease the cost of acquiring data and also made the classification model easier to understand by using the set of typical testors, taking out irrelevant or redundant features, reducing the number of features.

However, accurate recognition for normal and HCC nuclei still remains a significant challenge because of two main reasons. One is that since hematoxylin and eosin (H&E) pathology images vary distinctly in color, this may reduce the effectiveness of most texture features. The other one is that precise morphological measurements of each nucleus require accurate nucleus segmentation as an important prerequisite. To address these issues, this paper proposes two novel kinds of features for the recognition between normal and hepatocellular carcinoma (HCC) nuclei. In contrast to other methods that used features of different image channels, our goal is to classify normal and HCC nuclei based only on binary results of nuclei. Besides, for nucleus segmentation accurately, center-proliferation segmentation (CPS) method [10] is utilized to segment each individual nucleus. The main contributions of this paper are as follows. First, nucleus shape library is constructed based on manual selection by pathologists, which contains normal and HCC nuclei. Then, all nuclei are adjusted into a uniform standard space with same center, area, and orientation. Dice, Jaccard, precision, and recall coefficients are calculated for measuring the shape similarity among nuclei. Second, 12 boundary feature points are determined according to the same interval angle, 220 different triangles are thus formed via every three points. The calculation of boundary similarity is implemented by considering the similarity of triangles. The final experiment results show that the two kinds of features combined with conventional random forest (RF) classifier could achieve satisfactory effect in terms of accuracy (ACC), sensibility (SEN), and specificity (SPE).

## 2. Related Basic Knowledge

### 2.1. Features

Cell image features are one of the most obvious attributes for classification. Good features can not only influence the performance of classification but also improve the accuracy. Three kinds of characteristics are presented in Table 1. Intensity features are mainly obtained by computing the pixel value of the whole image [14]. Morphology features express the spatial relative position of each pixel [15]. Texture is the most important group of features for classification [16–18].

### 2.2. Random Forest Classifier

**Figure 1:**
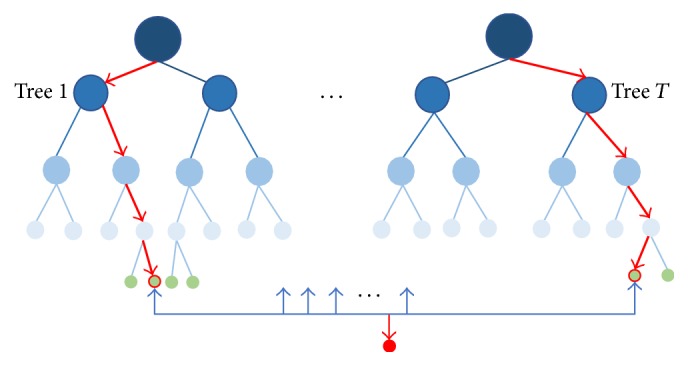
The flowchart of RF.

Random forest (RF) [19] is a joint prediction model composed of multiple decision tree, which can be used as an efficient and effective classification model. The principle of the classifier is to build a forest consisting of multiple decision tree with no association randomly. When a new sample comes, data utilize bootstrap method to extract in row and column and judge by every decision tree in the forest to see whether it belongs to this class or not. Final predict result generates by voting. As shown in Figure 1, the flowchart of RF is described.

RF increases the diversity between two classification models by constructing different training sets and holds the advantages of handling overfitting and resisting noise. After training, every decision tree model has its voting rights to choose the classification result as shown in the following formula:

(1) $$\begin{array}{c} H\left(\;x\;\right)=\underset{Y}{max}\overset{k}{\underset{i=1}{\sum }}I\left(\;h_{i}\left(\;x\;\right)=Y\;\right), \end{array}$$

where *H*(*x*) indicates the final classification result, *h*_*i*_(*x*) is the classification results of each decision tree, *Y* is the output target, and *I*(·) is indicative function.

## 3. Material and Method

### 3.1. Dataset Description and Nucleus Segmentation

**Figure 2:**
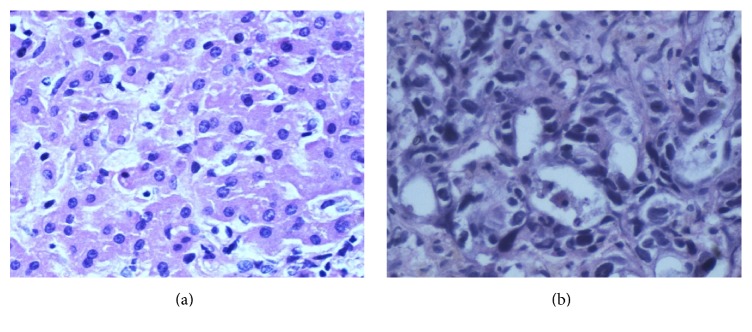
The H&E pathology images. (a) A normal pathology image. (b) A HCC pathology image.

Experimental data coming from a renowned hospital in Shenyang, China. A hundred and twenty-seven (127) liver pathology cases and labels consisting of the images are given under the supervision of the professional pathologists. Throughout the discussions with the pathologists, the concrete processes of obtaining the experimental data are as follows. Firstly, tissue slices are acquired through paraffin-embedding. Then, the H&E slides are cut at 4 *μ*m thickness by a microtome and stained using hematoxylin and eosin for 7.5 min. Finally, fast slide scanners are used to generate digital images for image capture at 20x magnification. In addition, each image's resolution is 0.35 mm/pixel.  Figure 2 shows the normal and HCC pathology images.  Figure 2(a) is a normal image and Figure 2(b) is a HCC image.

Our intention of this paper is to extract the morphological features which include shape and boundary similarity features; we thus employ center-proliferation segmentation (CPS) to obtain the segmentation result of each nucleus. The specific steps are introduced as follows.

**Step 1 .**

Choose a suitable threshold to get the binary nucleus coarse segmentation results and receive the connected region according to coarse segmentation results.

**Step 2 .**

Select the connected region by computing the circularity and set the threshold to a value larger than 0.85.

**Step 3 .**

Locate and acquire the center of each nucleus through the selected connected region and map the centers' coordinates into the corresponding H&E pathology images.

**Step 4 .**

Acquire *n* × *n* pixel nucleus patches from each center to the four directions.

**Figure 3:**
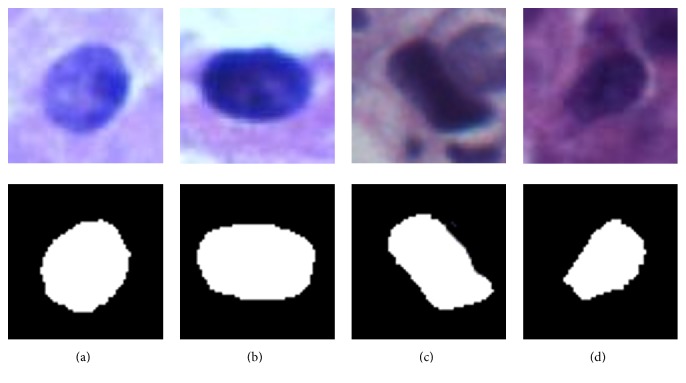
The H&E nucleus and the corresponding binary patches. The first row shows four H&E nuclei. The second row indicates the corresponding binary patches.

Following this method, the segmentation result of each nucleus is acquired. For the reason of guaranteeing every nucleus in the well-distributed scale position of the images, we resize each image to 100 × 100 pixels in the experiments.  Figure 3 shows the examples of nucleus image patches. The first row is the segmented patches of H&E image and the second row represents the corresponding binary segmentation results. To alleviate the influence of color difference, our experiments only utilize the binary patches to extract the morphological features.

### 3.2. Methods

**Figure 4:**
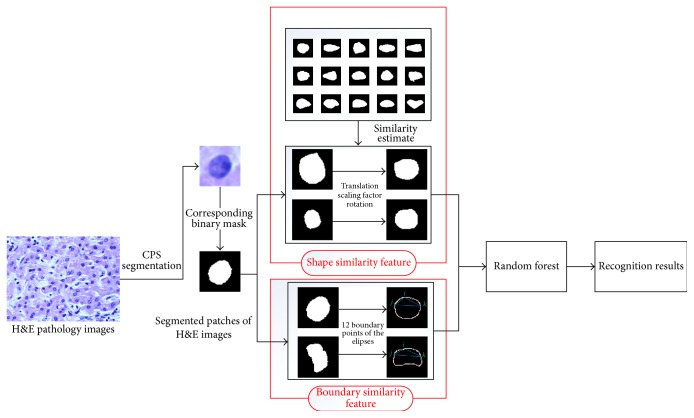
General overview of our nucleus recognition framework.

The diagram of our proposed nucleus recognition framework is shown in Figure 4. It first decomposes an H&E pathology image into some patches with the fixed size using the CPS method, which satisfy that one patch contains one nucleus. Then, the corresponding binary masks are accordingly obtained via morphological operations. Next, two novel kinds of features, shape similarity feature (Section 3.2.2) and boundary similarity feature (Section 3.2.3), are extracted to construct the feature vectors. Finally, conventional random forest (RF) is used to recognize normal nucleus or HCC nucleus.

#### 3.2.1. Classification Steps

**Figure 5:**
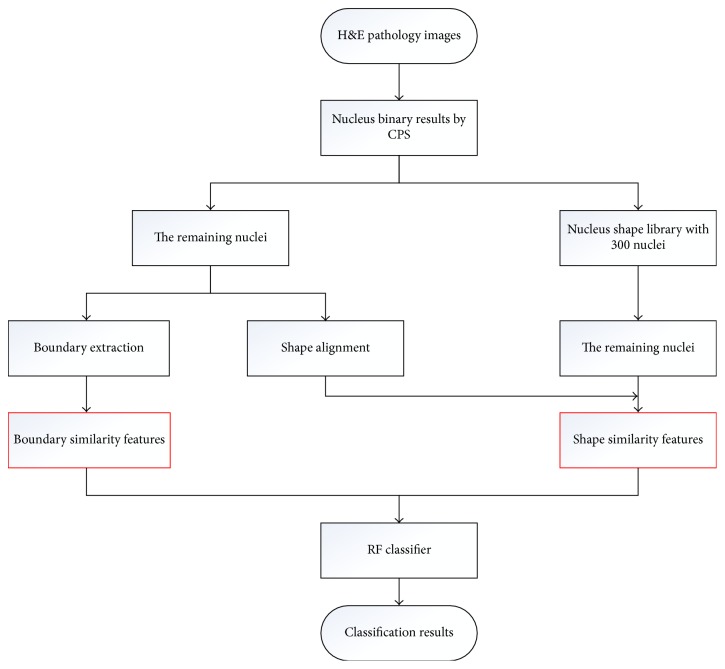
The flowchart of the proposed HE pathological image classification method.

As shown in Figure 5, the proposed classification model is described, which composed of the following steps. Note that the red rectangular frames represent the innovation work of this paper.

**Step 1 .**

For each liver pathology image, the center-proliferation segmentation (CPS) method [10] is utilized to obtain the segmentation result (binary result) of each individual nucleus. For the sake of effective image processing, each segmented nucleus is located at the same size of patch with 100 × 100 pixels.

**Step 2 .**

160 segmented nuclei are manually selected to construct the nucleus shape library under the guidance of the pathologists, which contain 80 normal nuclei and 80 HCC nuclei, respectively. Then, a shape alignment method is employed to adjust these nuclei into a uniform standard space.

**Step 3 .**

In order to calculate shape similarity accurately, the remaining nuclei are also adjusted into the uniform standard space with the same center, area, and orientation. Dice, Jaccard, precision, and recall indexes are calculated between each nucleus and all nuclei of shape library. Based on these indexes, shape similarity features for each nucleus are formed with 640 dimensions in total.

**Step 4 .**

According to the major axis and minor axis' lengths of each original segmented nucleus, the corresponding ellipse is obtained and we select 12 boundary points with the same interval (*π*/6). The initial point is defined at the positive direction of the horizontal axis and the rotational direction is anticlockwise. Following these 12 points of each ellipse, 12 boundary feature points of each corresponding nucleus are determined using the minimum value of Euclidean distance between each boundary point and 12 ellipse points. Every 12 boundary points could construct 220 different triangles and we use the similarity between triangles to measure boundary similarity features. Notice that boundary similarity features for each nucleus are 220 dimensions in total.

**Step 5 .**

Finally, the conventional RF classifier is used to train and test all nucleus images. In order to remove the redundancy of the features, 10-fold cross-validation experiments show that combining the selected boundary similarity features (56-dimension) with Jaccard shape similarity features (80-dimension) into the classifier could achieve the best results in terms of ACC, SEN, and SPE.

#### 3.2.2. Shape Similarity Feature

Generally, nuclei with the same label (normal and HCC) are similar in shape, boundary, and appearance. A basic approach is to use normal and HCC nuclei to establish statistical shape models and then measure the difference to classify a nucleus as normal or HCC. However, the statistical shape model for each class is unreliable. To improve this issue, we select 160 nuclei to construct a nucleus shape library under the guidance of the pathologists, which include 80 normal nuclei and 80 HCC nuclei, respectively. Our intention in this section is to extract the shape similarity features using the similarity measurement between each nucleus and all nuclei of the shape library. Further, in order to reduce the influence of shape difference caused by translation, rotation, and scale, a simple registration method is performed on each segmented individual nucleus into a uniform standard space. A new point *a*′ is located using the transformation:

(2) $$\begin{array}{c} a^{\prime }=\sigma Ra+T, \end{array}$$

where *a* and *a*′ are an original point and the corresponding transformation point, respectively. *σ* is the scaling factor, *R* is the rotation matrix, and *T* denotes the translation vector. In our experiments, each nucleus needs to be adjusted to the uniform standard space defined as follows.

*Translation T*. Align each nucleus centroid to patch center and calculate coordinate translation vector:

(3) $$\begin{array}{c} \left(\;\Delta x_{i},\Delta y_{i}\;\right)=\left(\;x_{c},y_{c}\;\right)-\left(\;x_{i},y_{i}\;\right), \end{array}$$

where (Δ*x*_*i*_, Δ*y*_*i*_) represents the corresponding coordinate translation vector between patch center (*x*_*c*_, *y*_*c*_) and each nucleus centroid (*x*_*i*_, *y*_*i*_).

*Scaling Factor σ*. Zoom or shrink all nucleus areas to 1000 pixels approximately:

(4) $$\begin{array}{c} \sigma_{i}=\frac{1000}{area_{i}}, \end{array}$$

where *σ*_*i*_ is the scaling factor and area_*i*_ is each nucleus area represented by the number of pixels.

*Rotation Matrix R*. Adjust the principal axes of all nuclei into the horizontal axis:

(5) $$\begin{array}{c} R\left(\;\theta \;\right)=\left[\begin{array}{cc} \cos\left(\theta \right) & -\sin\left(\theta \right)\\ \sin\left(\theta \right) & \cos\left(\theta \right) \end{array}\right], \end{array}$$

where *θ* denotes an angle between the principal axe of each nucleus and the positive direction of horizontal axis.

**Figure 6:**
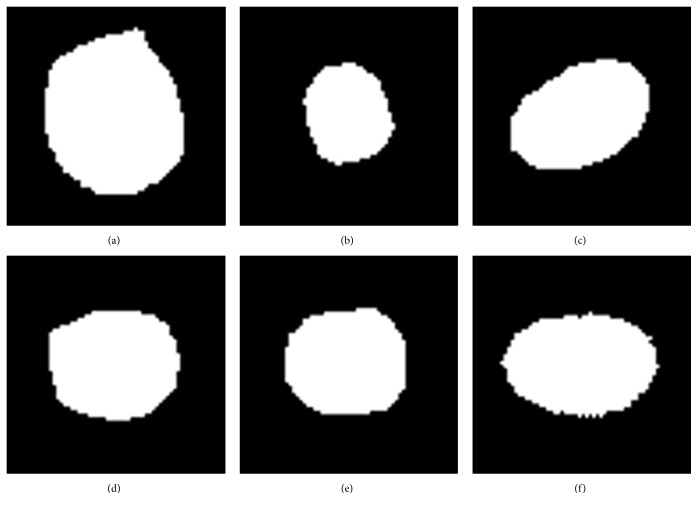
The examples of nucleus alignments. (a), (b), and (c) Three segmented nuclei. (d), (e), and (f) The corresponding registration results.

Following this method, all segmented individual nuclei are adjusted into the uniform standard space of the same center, area, and direction. Some examples illustrating this alignment method are shown in Figure 6. Figures 6(a), 6(b), and 6(c) are the segmented nuclei. Figures 6(d), 6(e), and 6(f) present the corresponding registration results.

Next, four commonly used similarity metrics are considered:

(6) DISR,TR=2×NumpixelSR∩pixelTRNumpixelSR+NumpixelTR

(7) JISR,TR=NumpixelSR∩pixelTRNumpixelSR∪pixelTR

(8) PSR,TR=NumpixelSR∩pixelTRNumpixelSR

(9) RSR,TR=NumpixelSR∩pixelTRNumpixelTR,

where Num(·) represents the number of all pixels in the nucleus regions. pixel_SR_ and pixel_TR_ are the pixels in the segmentation regions (SR) and ground truth regions (TR), respectively. DI(SR, TR) is Dice index which describes the similarity degree directly. JI(SR, TR) denotes Jaccard index that measures the difference between the segmentation results and ground truth. P(SR, TR) and R(SR, TR) represent precision and recall, respectively.

Finally, the shape similarity features for each nucleus are formed by calculating (6), (7), (8), and (9) with all nuclei of shape library. Obviously, the number of features is the same as the number of nuclei of the shape library. In this paper, we denote DI, JI, P, and R shape similarity features as DI feature, JI feature, P feature, and R feature, respectively. It can be seen that these four kinds of shape similarity features are 160 dimensions, respectively.

#### 3.2.3. Boundary Similarity Feature

It is our understanding that there is a wealth of geometric information with regard to the boundary of an object. Many classification tasks using boundary information could achieve remarkable achievements, such as the average value or standard deviation between the distances of all boundary points and the center point, and this can be regarded as a kind of statistical characteristics. The other distinguished boundary feature is concave-convex, which has demonstrated significant superiority via measuring monotonicity variation of the boundary curve. However, it is cumbersome to express the curve analytically due to its irregularity. To address this issue, we propose a novel boundary similarity feature using the triangles formed by the boundary points. The concrete steps are as follows.

**Step 1 .**

The Canny operator is utilized to extract the boundary for each nucleus. Note that, for the sake of accurate calculation, all nuclei used for boundary similarity feature need to be adjusted into the same center and direction using (3) and (5).

**Step 2 .**

For each nucleus, the corresponding ellipse is delineated via the nucleus' major axis and minor axis. 12 points of ellipse boundary are determined through different polar angles with the same interval. Hence, the points' coordinates could be calculated as follows:

(10) x=acos⁡θy=bsin⁡θ,

where *a* and *b* represent the major axis and minor axis' lengths of each nucleus, respectively. (*x*, *y*) is the Cartesian coordinates. *θ* denotes the polar angles and we define that the initial angle's value is from 0 to *π*/6. The angle interval is fixed as *π*/6.

**Step 3 .**

Then, we calculate the Euclidean distance between each nucleus boundary point and the corresponding 12 points of ellipse boundary. 12 boundary feature points of each nucleus are determined according to the minimum values of Euclidean distance.

**Step 4 .**

Based on these 12 boundary feature points for each nucleus, we could construct 220 different triangles. This section proposes to represent the boundary similarity features of a nucleus by measuring the angles of the 220 triangles. Specifically, given a triangle containing three control points *i*, *j*, and *k*, the shape of the triangle could be represented by storing just two of its angles (e.g., *∠ijk* and *∠ikj*) since the sum of three sides of a triangle is equal to *π*. Finally, the boundary similarity feature could be calculated in the following:

(11) $$\begin{array}{c} BF=\frac{\cos\left(∠ijk\right)}{\cos\left(∠ikj\right)}, \end{array}$$

where BF represent the boundary similarity feature and we can see that the number of this feature is 220 dimensions.

**Figure 7:**
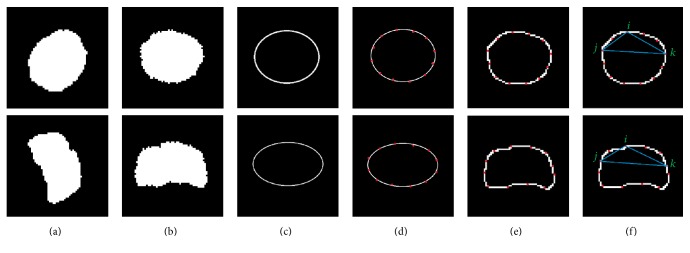
The process of extracting the boundary similarity features for each nucleus. (a) Two segmented nuclei. (b) The registration nuclei with the same center and direction. (c) The corresponding ellipse templates. (d) 12 boundary points of the ellipses. (e) The determined boundary feature points for each nucleus. (f) A triangle formed by three corresponding different control points named *i*, *j*, and *k*, respectively.

Following this approach, the boundary similarity features for each nucleus are obtained and two examples are shown in Figure 7.  Figure 7(a) are two segmented nuclei.  Figure 7(b) are the registration nuclei with the same center and direction.  Figure 7(c) are the corresponding ellipse templates.  Figure 7(d) show the 12 boundary points of the ellipses.  Figure 7(e) are the determined boundary feature points for each nucleus.  Figure 7(f) presents a triangle formed by three corresponding different control points named *i*, *j*, and *k* respectively.

So far we have considered the complete feature set for each nucleus as only combining shape and boundary similarity features. In our case, the conventional support vector machine (SVM), extreme learning machine (ELM), and RF are utilized to train and test all feature data.

## 4. Results and Comparisons

### 4.1. Experimental Data and Platform

Experimental data containing 9720 image patches are obtained using the method described in Section 3.1. The number of training patches and testing patches is shown in Table 2. The experimental platform is Intel(R) Core(TM) i7-4790 CPU@3.60 GHz, 8 G RAM, 930 G hard disk, Windows 7 OS, and MATLAB R2016a simulation environment.

### 4.2. Experimental Evaluative Criteria

In order to verify the proposed method for HCC image classification, three commonly used evaluative criteria are considered as

(12) ACC=TP+TNTP+FN+TN+FPSEN=TPTP+FNSPE=TNTN+FP,

where TP and FN are the number of HCC image patches which are correctly classified and incorrectly classified, respectively. TN and FP are the number of normal image patches which are correctly classified and incorrectly classified, respectively. ACC is the overall classification accuracy. Sensitivity (SEN) indicates the proportion of HCC image patches that are correctly classified and specificity (SPE) indicates the proportion of normal image patches that are correctly classified.

### 4.3. Results

**Figure 8:**
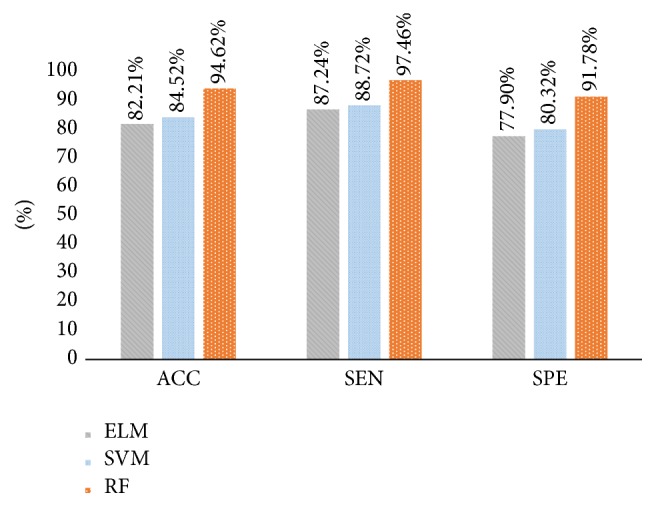
Average ACC, SEN, and SPE results using all shape and boundary similarity features with ELM, SVM, and RF.

**Figure 9:**
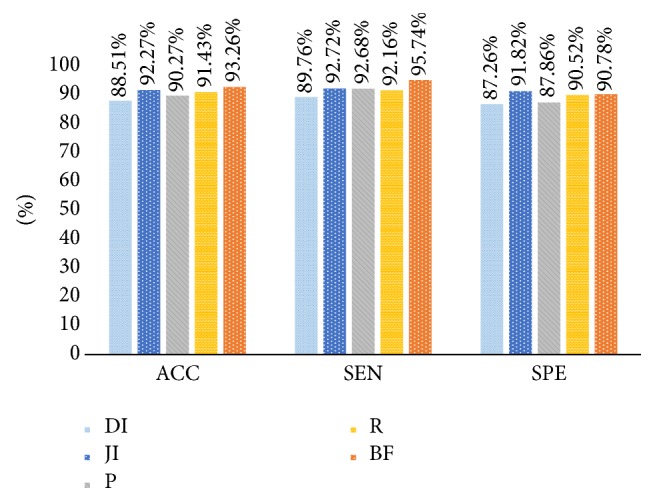
Average ACC, SEN, and SPE results using DI, JI, P, R, and BF features with RF, respectively.

**Figure 10:**
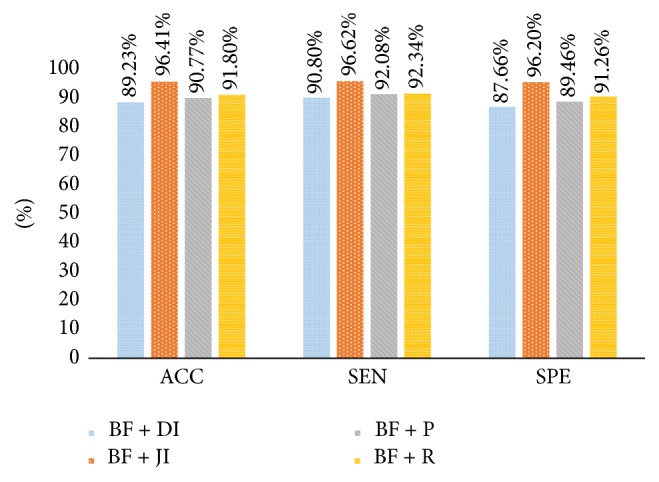
Average ACC, SEN, and SPE results using BF + DI, BF + JI, BF + P, and BF + R feature combinations with RF, respectively.

**Figure 11:**
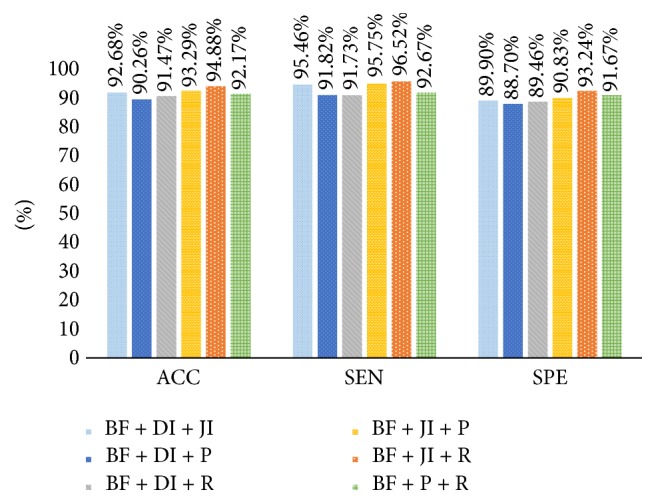
Average ACC, SEN, and SPE results using BF + DI + JI, BF + DI + P, BF + DI + R, BF + JI + P, BF + JI + R, and BF + P + R feature combinations with RF, respectively.

**Figure 12:**
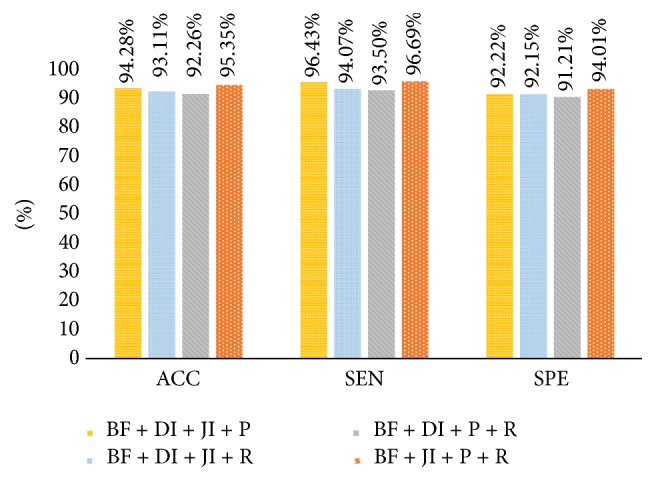
Average ACC, SEN, and SPE results using BF + DI + JI + P, BF + DI + JI + R, BF + JI + P, and BF + JI + P + R feature combinations with RF, respectively.

Performance results of shape and boundary similarity features are presented in this section. Note that we test our method 10 times and randomly select the training and testing data according to Table 2 every time.  Figure 8 shows average HCC or normal classification ACC, SEN, and SPE using all shape and boundary similarity features (860 dimensions for each nucleus) when three types of classifier are used, including RF, SVM, and ELM. It is seen that these features with RF classifier perform best. Further, 5 features (DI, JI, P, R, and BF features) are utilized to train and test RF classifier.  Figure 9 presents the corresponding average classification ACC, SEN, and SPE. We can see that the BF feature could achieve the best effect. In addition, to find the best feature combination for accurate classification, we also combine different shape and boundary similarity features to train and test RF classification. Figures 10, 11, and 12 show the corresponding ACC, SEN, and SPE results. In conclusion, Figures 8, 9, 10, 11, and 12 demonstrate that JI + BF with RF classifier is much better than other features or feature combinations.

### 4.4. Feature Selection Results

**Figure 13:**
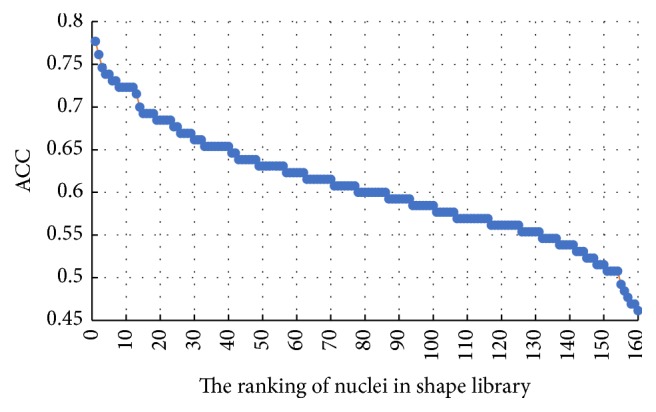
The average ACC results for each nucleus in shape library.

**Figure 14:**
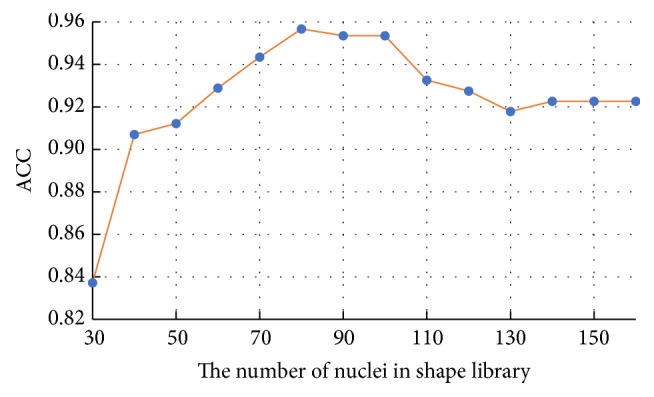
The average ACC results for different number of shape library's nuclei.

**Figure 15:**
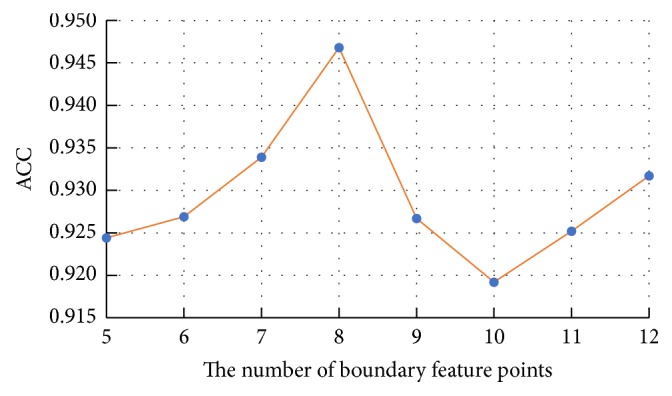
The average ACC results for different number of boundary feature points.

Generally, the number of nuclei in shape library and the number of boundary feature points are determined by experience. However, this may generate some redundancy and reduce the classification accuracy. To address this issue, 10-fold cross-validation and grid-search technology are adopted in our experiments to select the number of shape library's nuclei and boundary feature points. We first test the performance of each nucleus of shape library separately. The ACC results of each nucleus ranking from high to low are shown in Figure 13. Next, we integrate the first 30 nuclei as the new shape library and add 10 nuclei into the shape library for training and testing successively. Finally, the corresponding average ACC results for different number of shape library's nuclei are presented in Figure 14. Obviously, the shape library composed of the first 80 nuclei is the most beneficial for HCC nucleus recognition. For the boundary similarity feature, 12 boundary feature points constitute 220 different triangles and we also test the performance of each triangle separately. According to the individual test results, the importance of 12 boundary feature points ranking from high to low is determined through the occurrence number of each feature point in the triangles.  Figure 15 shows average ACC results, which includes the different boundary similarity feature sets formed by corresponding 5, 6, 7, 8, 9, 10, 11, and 12 boundary feature points. It is seen that 8 boundary feature points could achieve the best performance with regard to boundary similarity feature. To sum up, 136-dimension features, constructed via 80 shape similarity features (80 nuclei in shape library) and 56 (*C*_8_^3^) boundary similarity features (8 boundary feature points), are considered as the optimal feature set for our recognition task.

### 4.5. Comparisons

**Figure 16:**
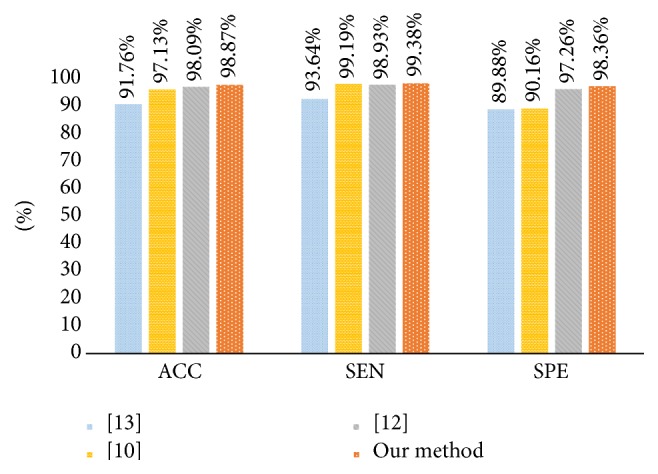
Average ACC, SEN, and SPE results with different literatures [10, 12, 13] and our method.

In order to further evaluate the proposed method with other related work, three methods in [10, 12, 13] are performed on our data for fair comparison. The intention of [10] is to propose some atypia features (auxiliary circularity, amendment circularity, and cell symmetry) and a voting ranking random forest to establish the classification model. The core of [12] is to utilize the concave-convex variations of nucleus boundaries to improve the performance of each classifier. Finally, the intention of [13] is to propose a simple but robust local descriptor without any quantization for local patch representation.  Figure 16 presents the averaging ACC, SEN, and SPE results using 10-fold cross-validation. Note that comparison experiments utilize the optimal 136-dimension feature set and we can see that our method is slightly superior to [10, 12, 13].

### 4.6. Shape Library Selection

As previously mentioned, the nucleus shape library is composed of 160 nuclei including 80 normal nuclei and 80 HCC nuclei, which are selected by the pathologists. To further reduce manual process, we randomly select 10 groups of nuclei as the shape library and the remaining nucleus patches are used to examine the effect of different nucleus shape library. Similarly, each group of nuclei contains 80 normal nuclei and 80 HCC nuclei. The results for different shape libraries are presented in Table 3 and an interesting finding is that random selection of nucleus shape library influences little final classification results.

## 5. Discussions

For our shape similarity feature, different similarity measures are utilized to establish the feature set. According to Figure 9, Jaccard index is regarded as the JI features, which could achieve more effective classification results than other similarity measurements. This shape similarity feature measures the similarity and difference between each nucleus and all nuclei of shape library. The shape registration method is utilized to adjust all nuclei into the same center, scale, and direction and then we can obtain more accurate shape similarity features. Besides, Table 3 presents the effects of different shape libraries. It is our understanding that random selection for different shape libraries influence little final classification results and this feature could thus be considered as a robust feature. In regard to our boundary similarity feature, Figure 9 shows that BF feature could achieve relatively ideal results. BF feature is calculated via the similarity of triangles constructed by boundary feature points and the boundary feature points are determined by corresponding ellipse template. Different from other similarity measurement of curves, this calculation method is simple and effective. In addition, Figure 10 demonstrates that JI + BF feature combination with RF classifier perform best and this paper treats JI + BF as the optimal feature combination. Further, we determine the optimal number of JI + BF features using 10-fold cross-validation (see Figures 13, 14, and 15). Finally, Figure 16 shows that our proposed method overcomes other related methods in terms of ACC, SEN, and SPE, demonstrably the performance superiority of our proposed two morphological features.

## 6. Conclusions

In this paper, we propose two novel kinds of features for normal and hepatocellular carcinoma (HCC) nucleus recognition, including shape and boundary similarity. First, the shape similarity feature is extracted via the Jaccard index's calculation between each nucleus and all nuclei of the shape library. Then, the boundary similarity feature is computed through the similarity of triangles constructed by boundary feature points. Next, combining JI and BF features (136-dimension) is regarded as the feature set for image patches. Finally, the conventional RF classifier is used for obtaining the best classification results. Experiments with 9720 patches demonstrate that our proposed morphological features (JI + BF) with RF classifier are beneficial and robust to achieve the satisfactory results in terms of ACC, SPE, and SEN.

## Figures and Tables

**Table 1:** Three kinds of characteristics.

Category	Features
Intensity	Density, mean, median, variance, kurtosis, skewness, and so forth
Morphology	Area, perimeter, diameter, area overlap ratio, center of mass, minor axis, major axis, smoothness, symmetry, concavo-convex, and so forth
Texture	Gray level cooccurrence matrix, local binary pattern, scale-invariant feature transform Tamura, fractal, Markov random field, wavelets, Haar-like features, Gabor, run-length, and so forth

**Table 2:** The number of training images and testing images used in our experiment.

Patch type	Training patches	Testing patches	Total patches
Normal patches	2600	2260	4860
HCC patches	2600	2260	4860
Total patches	5200	4520	9720

**Table 3:** The ACC, SEN, and SPE effects for different shape libraries.

Group number	ACC	SEN	SPE
1	93.94%	96.23%	91.65%
2	96.09%	95.46%	96.72%
3	93.89%	96.07%	91.71%
4	92.93%	94.33%	91.53%
5	92.94%	95.17%	90.71%
6	93.77%	96.89%	90.65%
7	91.75%	97.23%	86.27%
8	91.88%	96.78%	86.98%
9	94.95%	94.78%	92.42%
10	93.88%	95.06%	92.80%
